# Genital Schistosomiasis: A Report on Two Cases of Ovarian Carcinomas Containing Viable Eggs of *Schistosoma mansoni*

**DOI:** 10.1155/2014/508718

**Published:** 2014-12-22

**Authors:** Andressa Gonçalves Amorim, Fernanda Alves Barbosa Pagio, Rodrigo Neves Ferreira, Antônio Chambô Filho

**Affiliations:** ^1^Department of Obstetrics and Gynecology, Santa Casa de Misericórdia Hospital, 143 Dr. João dos Santos Neves, 29025-023 Vitória, ES, Brazil; ^2^Department of Pathology, Santa Casa de Misericórdia Hospital, 143 Dr. João dos Santos Neves Street, 29025-023 Vitória, ES, Brazil

## Abstract

Schistosomiasis is a parasitic infection that is highly prevalent worldwide, with a variety of species being responsible for causing the disease. In Brazil, however, the only identified species is *Schistosoma mansoni*. The adult parasites inhabit the blood vessels of the hepatic portal system of the main host. The disease may range from being asymptomatic to provoking liver damage or portal hypertension. Furthermore, ectopic schistosomiasis may develop, and several hypotheses have been raised to explain the occurrence of the disease. This paper describes two cases, one in a 39-year-old woman and the other in a 47-year-old woman. Both had similar symptoms of pain and abdominal distension caused by a large abdominal/pelvic mass. Histopathology of the ovary showed a mucinous cystadenocarcinoma of the intestinal type in the first patient and a papillary serous carcinoma in the second, with both tumors containing viable eggs of *Schistosoma mansoni*. The neoplasms probably serve as a migratory route for the adult parasites and the embolization of eggs. Nevertheless, there is insufficient evidence to confirm the malignization of a benign lesion due to the presence of *Schistosoma mansoni*. Few cases have been reported in the international literature on the association between ovarian schistosomiasis and neoplasms.

## 1. Introduction

Ovarian epithelial carcinoma is the second most common cancer of the female genital tract. It corresponds to 5% of all female tumors [1] and is the third cause of death from cancer in women [2].

Protective factors for ovarian cancer include parity, breastfeeding, the use of oral contraceptives, late menarche, early menopause, and surgical procedures such as hysterectomy and salpingectomy. Risk factors include the use of ovulation-inducing drugs, high estrogen and androgen levels, and genetic and environmental factors [3].

In rare cases, an association has been reported between neoplasms and schistosomiasis, with studies showing that the ovaries are affected in only 0.5% of cases of ectopic schistosomiasis [4].

Schistosomiasis caused by* S. mansoni* is endemic in the Caribbean islands, Middle East, South America, and Africa and can be imported to any region of the world through immigration [5]. The disease is considered a severe public health issue [6]. Between 200 and 300 million individuals are believed to be infected with* S. mansoni* worldwide. In Brazil, this figure is estimated at 4 to 6 million individuals [7]. Currently, schistosomiasis is present in almost all the Brazilian states, principally in areas of the northeastern, southeastern, and midwestern regions [8]. Various species of* Schistosoma* cause schistosomiasis; however, only* S. mansoni* has been reported in Brazil [9].

This parasitic infection is an important cause of morbidity and mortality and has been found in various organs of the body. There is generally no clinical suspicion of schistosomiasis in these lesions; indeed, in the majority of cases, the infection is only discovered by chance at histopathology [10].

Two cases are reported here in which histopathology identified viable* Schistosoma mansoni* (*S. mansoni*) eggs associated with an ovarian mucinous cystadenocarcinoma of the intestinal type (case #1) and with a papillary serous carcinoma (case #2) in patients receiving care at the Department of Obstetrics and Gynecology, Santa Casa de Misericórdia Hospital, Vitória, Espírito Santo, Brazil. The internal review board approved this report under reference number 25471214.0.0000.5065. Both patients gave their signed informed consent to the publication of the images that form part of this paper.

## 2. Case Report #1

This patient, a 39-year-old woman, was hospitalized complaining of progressive abdominal distension and edema of the lower limbs over the previous month. She also complained of abdominal pain and frequent micturition. There were no signs of dysuria, gastrointestinal symptoms, fever, loss of appetite, or weight loss.

Physical examination showed the patient to be in an apparently good general state of health. She was well hydrated, her temperature was normal, and there were no signs of anemia, jaundice, or cyanosis. No cardiovascular or respiratory abnormalities were found. The distended abdomen presented normal bowel sounds, with a large, hardened, irregular mass extending from the pelvis to the epigastrium. Palpation of the liver and spleen revealed no abnormalities. There were no signs of ascites or enlarged lymph nodes. Gynecological examination revealed an apparently normal cervical epithelium and external os with no abnormal discharge. Digital pelvic examination showed a fibroelastic, closed cervix that was mobile and painless. Bimanual palpation revealed a large pelvic mass protruding into the Douglas pouch. The borders of the uterine fundus were unclear due to the size of the tumor.

Pelvic ultrasonography revealed a large abdominal/pelvic mass of 6,606.7 cm³, with a predominantly cystic appearance and a solid component. There was an anechoic cystic mass of 4 cm³ in volume on the right ovary. Uterine volume was 180 cm³ and endometrial thickness was 6.7 mm. There were no signs of ascites. Doppler flow showed a low resistance index of 0.44.

The results of supplementary tests showed CA-125 levels of 24.87 U/mL, hemoglobin 10.5 g/dL, hematocrit 35.6%, mean corpuscular volume (MCV) 81.2 fl, mean corpuscular hemoglobin (MCH) 27.5 pq, mean corpuscular hemoglobin concentration (MCHC) 32.2%, and coefficient of variation of red cell distribution width (RDW-CV) 12.0%. White blood cell count was 8,500/cm³, with 3% eosinophils (255/mm³).

### 2.1. Surgery

Exploratory laparotomy performed through a midline infraumbilical incision showed a large, predominantly cystic tumor occupying the entire abdominal/pelvic cavity, with its epicenter in the left ovary. Extraovarian lesions and signs of ascites were then noted. A hysterectomy was performed, with bilateral adnexectomy, partial omentectomy, and pelvic/aortic lymphadenectomy. The patient progressed satisfactorily with no postoperative complications and was discharged from hospital.

### 2.2. Histopathology of the Surgical Specimen

**Figure 1:**
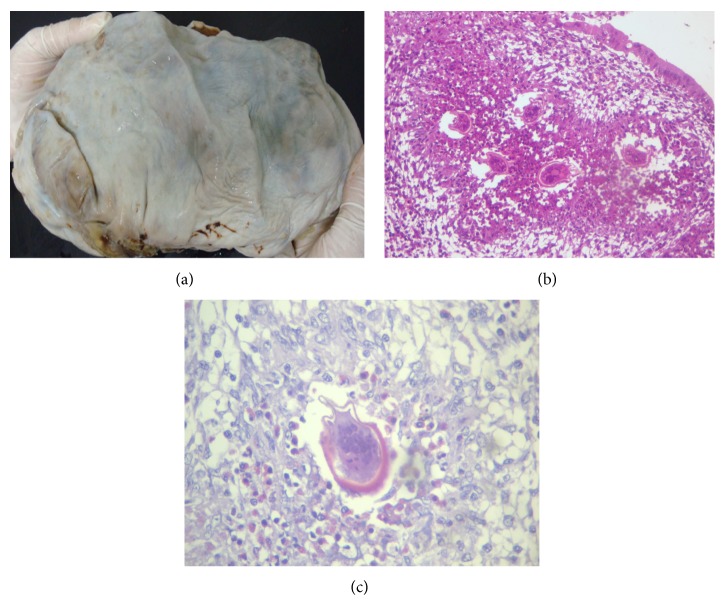
Case report #1. (a) Macroscopic view of the left ovary measuring 25 × 20 × 8 cm. (b) Viable* S. mansoni* eggs (hematoxylin-eosin, magnification 40x). (c) Detailed view of a* S. mansoni* egg (periodic acid-Schiff, magnification 400x).

Cytology was positive for malignant cells. The uterus had no notable histopathological abnormalities. Macroscopically, the left ovary measured 20 × 20 × 8 cm, with a smooth, shiny surface (Figure 1(a)). Sectioning revealed a cystic lesion with irregular internal walls, friable vegetation, and solid areas of white tissue. Microscopic examination revealed a mucinous cystadenocarcinoma of the intestinal type with solid, cystic, cribriform, and infiltrating areas with foci of necrosis. Numerous viable* S. mansoni* eggs surrounded by eosinophilic granulomas permeating the neoplastic tissue were found in 2 of the 30 slides examined (Figures 1(b) and 1(c)). Reactive inflammation and the fact that the eggshells were intact indicated that the eggs were viable. There was no fibrosis or calcification.

None of the clinical or complementary examinations to which the patient was submitted showed any signs of parasitic infection, except for the findings of* S. mansoni* eggs in the surgical specimen.

The patient was referred for adjuvant chemotherapy, and a single dose of praziquantel 50 mg/kg was prescribed to treat her schistosomiasis. She was referred to a center for parasitic infections and was not followed up at this clinic due to the fact that she lives in another state.

## 3. Case Report #2

This patient, a 47-year-old postmenopausal woman, was hospitalized due to progressive abdominal distension over the previous year. She also reported abdominal pain but no gastrointestinal symptoms, fever, and loss of appetite or weight. At the time of admission to hospital, the patient was complaining of a dry cough.

Physical examination showed the patient to be in an apparently good general state of health and well hydrated, with normal temperature and no signs of anemia, jaundice, or cyanosis. There were no signs of cardiovascular or respiratory abnormalities. The abdomen was distended, with normal bowel sounds and an irregular, voluminous mass extending from the pelvis to 15 cm above the umbilicus. Gynecological examination showed a hypotrophic cervix, epithelialization, slit-shaped external os with no apparent lesions, and the presence of a yellowish, malodorous secretion. Digital pelvic examination showed a closed, largely immobile cervix, with no pain at manipulation. Bimanual palpation revealed the presence of a large pelvic mass.

CT of the pelvis showed a large, expansive heterogeneous mass, with partially defined, irregular borders, multiple septa, and internal amorphous calcifications, with its epicenter in the pelvic cavity, predominantly to the right, with maximal longitudinal, anteroposterior, and transverse diameters of 20.0, 15.3, and 23.4 cm, respectively.

The results of supplementary tests showed CA-125 levels of 952.93 U/mL, hemoglobin 12.9 g/dL, hematocrit 37.7%, mean corpuscular volume (MCV) 83.2 fl, mean corpuscular hemoglobin (MCH) 28.5 pq, mean corpuscular hemoglobin concentration (MCHC) 34.2%, and coefficient of variation of red cell distribution width (RDW-CV) 13.0%. White blood cell count was 7,790/cm³, with 2% eosinophils (156/mm³).

### 3.1. Surgery

Exploratory laparotomy was performed through a midline infraumbilical incision, revealing a large pelvic mass of mixed composition occupying almost the entire abdominal/pelvic cavity, extending up to the hypochondrium, adhered to the uterus, adnexa, and bowel. Hysterectomy was performed and included bilateral adnexectomy, partial omentectomy, and pelvic/aortic lymphadenectomy. The patient progressed satisfactorily with no complications following surgery and was released from hospital.

### 3.2. Histopathology of the Surgical Specimen

**Figure 2:**
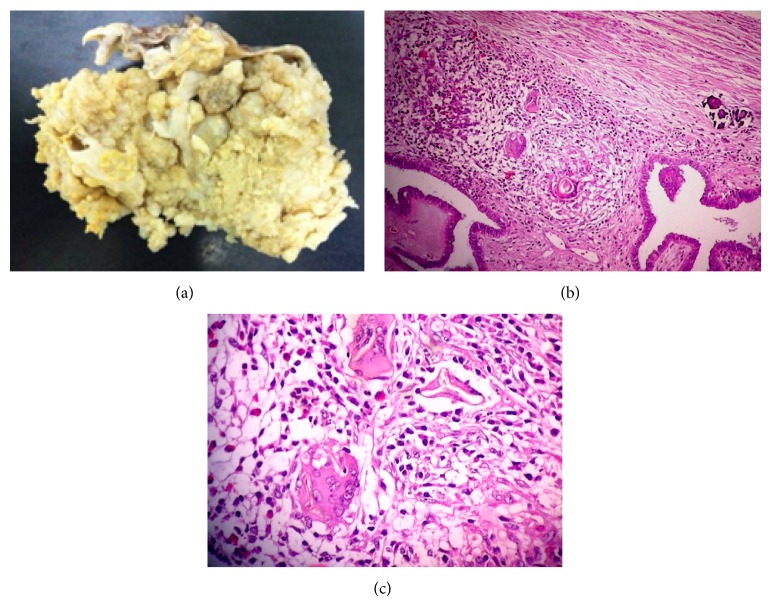
Case Report #2. (a) Macroscopic view of the right ovary measuring 21 × 17 × 13 cm. (b) Serous carcinoma with granulomas (hematoxylin-eosin, magnification 100x). (c) Viable* S. mansoni* eggs (hematoxylin-eosin, magnification 400x).

Cytology was positive for malignant cells. Macroscopically, the specimen measured 21 × 17 × 13 cm (Figure 2(a)) and weighed 1200 grams. The tumor consisted of various cysts, some with a semisolid content, and others were light brown in color and friable, infiltrating the capsule and exteriorizing part of the cyst. Part of the capsule of the cyst was thickened and hardened. The uterus measured 10 × 6 × 5 cm and weighed 115 grams, with a brownish serous membrane, fibrous adherences, and a brownish, friable mass in the region of the left ovary, which measured 8 × 8 cm. The histologic sections showed that the mass did not appear to be infiltrating the uterus. The endometrium was 0.2 cm thick and bleeding. The myometrium was 2 cm thick, with a small nodule measuring 0.4 × 0.3 cm. The left fallopian tube measured 4.5 × 1.3 cm, with a brownish serous membrane and patent lumen. A segment of the omentum measuring 25 × 8 × 0.6 cm was yellowish and firm/elastic. Microscopic examination of the right ovary detected a papillary serous carcinoma, grade 2/3, measuring 21 cm at its longest axis, with a solid papillary pattern. Tumor septate consisting of eosinophilic granulomas associated with viable* S. mansoni* eggs were found (Figures 2(b) and 2(c)). On the left ovary, a papillary serous carcinoma, grade 2/3, was present, measuring 8 cm at its largest axis. There were no signs of neoplasia in the uterus or in the fallopian tubes. In the omentum, there was metastasis from the multifocal serous carcinoma.

None of the clinical or complementary examinations to which the patient was submitted showed any signs of parasitic infection, except for the findings of* S. mansoni* eggs in the surgical specimen.

The patient was referred for adjuvant chemotherapy, and a single dose of praziquantel 50 mg/kg was prescribed to treat her schistosomiasis. She was followed up at a referral center for parasitic infections and by a clinical oncology specialist.

## 4. Discussion

Epithelial tumors originate in the epithelial surface and are classified at anatomopathology as serous, mucinous, clear-cell, endometrioid, or Brenner tumors [11]. Malignant ovarian tumors may be associated with high serum CA-125 glycoprotein levels (≥35 U/mL). However, this test is nonspecific, since the levels may also be high in the case of benign ovarian tumors, endometriosis, leiomyomas, and other diseases. Moreover, some patients with ovarian tumors may have low serum CA-125 levels [12]. As shown in the reports of these two cases, CA-125 levels were significantly increased in case #2 (papillary serous carcinoma), while in case #1 (mucinous cystadenocarcinoma of the intestinal type) the levels of CA-125 were normal.

The two cases reported above involved ovarian carcinomas, with the additional finding of* S. mansoni* eggs in the surgical specimens from the ovaries.

The biological cycle of schistosomiasis is complex and well defined, with water being the means through which the parasite infects the main host (vertebrate/man). In turn, contaminated human feces in contact with water spread the infection to the intermediate host (invertebrate—the* Biomphalaria* snail) [13]. The adult parasites live in the blood vessels that connect the bowel to the liver (the hepatic portal system) in the vertebrate host [14].

*S. mansoni* infestations are referred to as ectopic when the eggs or the adult form of the parasite is found outside the portal system, for example, in the skin, central nervous system, thyroid, myocardium, esophagus, stomach, gall bladder, pancreas, suprarenal, or urogenital tract [15]. These presentations may affect individuals with any parasite load, even years after the initial exposure [16]. The mechanism underlying ectopic migration remains to be clarified. The most widely accepted theory involves the migration of the adult parasites to the pelvic veins. Accordingly, the helminthes would travel in the opposite direction to that of blood flow, thus reaching the terminal portal venules. [17]. Other mechanisms that have been proposed are (a) embolization of the* S. mansoni* eggs through the network of arteries as a consequence of the presence of congenital cardiovascular defects or arteriovenous shunts and (b) egg-laying after the migration of the helminthes [17].

The transfer of eggs to pelvic region may generate reactions in the internal genital organs, leading to dyspareunia, vaginal discharge, bleeding, dysmenorrhea, lower abdominal pain, or abdominal/pelvic tumors, all of these being nonspecific findings often reported by gynecologists [18]. The vascularization of the female pelvis is peculiar in that it has numerous anastomoses that drain the female genital organs to the mesenteric venous system and consequently to the portal vein [14]. Some studies have shown that the microvascular network of preovulatory follicles is more developed and extensive compared to that of the other follicles, thus producing more substrates, nutrients, and trophic hormones that permit follicular development and growth. Therefore, vascularization in the female pelvis is distinctive, and the modifications that occur in angiogenesis during reproductive life and pregnancy may facilitate the implantation of ectopic schistosomiasis [19].

Neoplastic processes such as the tumors described here produce vascular endothelial growth factor (VEGF) and fibroblast growth factor (FGF), amongst other substances that permit the proliferation, survival, and differentiation of the endothelial cells, thus generating neovascularization, which is fundamental for the survival and progression of the neoplastic cells [20]. The neoplasms probably serve as a migratory route for the adult parasites and the embolization of eggs. The presence of viable* S. mansoni* eggs in the ovarian tumors described here strengthens this hypothesis. The presence of* S. mansoni* eggs causes inflammation and oxidative stress, which may lead to a poorer prognosis in the case of ovarian tumors. Nevertheless, there is insufficient evidence to confirm the malignization of a benign lesion due to the presence of* S. mansoni*. Further studies should be conducted in endemic areas to confirm this association, since in the majority of cases ectopic schistosomiasis lesions are diagnosed from surgical specimens or after death.

## 5. Conclusion

Studies on the association between* S. mansoni* and ovarian carcinoma are sparse in the literature; therefore, further studies should be conducted in endemic areas to provide further information on this association and on treatment in order to improve the current situation in which diagnosis in most cases is based on surgical specimens or reached following death.
